# Complications and Burden of 2-Stage Tissue Expander to Implant-Based Reconstructive Surgery: A Single-Center Retrospective Study

**DOI:** 10.1177/22925503231217517

**Published:** 2023-12-06

**Authors:** Isabella F. Churchill, Lucas Gallo, Emily Dunn, Cameron F. Leveille, Mark H. McRae, Ronen Avram, Sophocles H. Voineskos, Christopher J. Coroneos

**Affiliations:** 1 University of Ottawa, Faculty of Medicine, Ottawa, ON, Canada; 2 Division of Plastic Surgery, 3710 McMaster University, Hamilton, ON, Canada; 3 Department of Health Research Methods, Evidence, and Impact, 3710 McMaster University, Hamilton, ON, Canada; 4 Division of Plastic Surgery, University of Toronto, Toronto, ON, Canada

**Keywords:** breast surgery, complications, tissue expansion devices, 2-stage reconstruction, Chirurgie mammaire, complications dispositif d’expansion tissulaire, reconstruction en deux étapes.

## Abstract

**Introduction:** Two-stage reconstruction with a tissue expander/implant (TE/I) technique remains the most common breast reconstructive approach following mastectomy. This study analyzes the post-operative complications and burden associated with 2-stage TE/I reconstruction independent of acellular dermal matrix (ADM). **Methods:** A retrospective chart review identified patients that underwent 2-stage, reconstruction with TE/I without ADM. Demographics, implant characteristics, tissue expansion information, and complications were recorded. Patients were followed for 3 months post-implant exchange. Logistic regression analysis was used to determine which variables were predictors for complications. **Results:** Ninety-one TE/I reconstructions without ADM were performed in 55 patients. The incidence of complications was 45% (n = 25). Mean complications per patient was 0.84 ± 1.2, with the most common being infection with the TE (n = 15, 24.2%). Mean number of fill appointments was 3.6 ± 1.7 (range: 1-8). Univariate linear regression showed for every increase in BMI, there was a 14.8 cc increase in implant volume, on average (*P* < .001). Multivariable logistic regression model identified radiation history (*P* = .036) and increasing BMI (*P* = .049) as significant predictors for complications. **Conclusion:** Infection remains to be the leading cause of short-term complications in TE/I breast reconstruction patients. BMI and radiation are significant predictors. Larger, multicenter observational study data may elicit nuanced variation among different population demographics.

## Résumé

**Introduction :** La reconstruction en deux étapes avec une technique d’expanseur tissulaire/implant (ET/I) reste l’approche reconstructive du sein la plus courante après une mastectomie. Cette étude analyse les complications postopératoires et le fardeau associé à la reconstruction en deux étapes ET/I, indépendamment de l’ADM. **Méthodes :** Une étude rétrospective de dossiers a identifié les patients qui ont subi une reconstruction en deux étapes par ET/I sans ADM. Les données démographiques, les caractéristiques de l’implant, l’information sur l’expansion tissulaire et les complications ont été consignées. Les patients ont été suivis pendant trois mois après l’échange d’implant. Une analyse de régression logistique a servi à déterminer quelles variables étaient prédictrices de complications. **Résultats :** 91 reconstructions ET/I sans ADM ont été menées chez 55 patients. L’incidence des complications a été de 45% (n = 25). La moyenne des complications par patient a été de 0,84 ± 1,2, les plus fréquentes étant les infections avec l’ET (n = 15, 24,2%). Le nombre moyen de rendez-vous de remplissage a été 3,6 ± 1,7 (de 1 à 8). Une régression linéaire monofactorielle a montré que, pour chaque augmentation de l’IMC, il y avait une augmentation de 14 cc du volume de l’implant, en moyenne (*P* < 0,001). Le modèle de régression logistique multifactorielle a identifié les antécédents d’irradiation (*P* = 0,036) et l’augmentation de l’IMC (*P* = 0,049) comme étant des éléments prédictifs significatifs de complications. **Conclusion :** L’infection reste la principale cause des complications à court terme dans la reconstruction mammaire par ET/I. L’IMC et la radiothérapie sont des facteurs prédictifs significatifs. Les données d’une plus grande étude observationnelle multicentrique pourront faire apparaître des variations nuancées entre les différentes populations.

## Introduction

Breast cancer is the most common non-skin cancer affecting women in North America; 25,000 women in Canada are diagnosed annually.^1^ Recently, there has also been an increase in prophylactic contralateral mastectomies, with an associated rise in both immediate and 2-stage reconstruction.^2^ Today's demographic of breast reconstruction patient is often younger, more likely to be indicated for prophylactic or for an earlier stage of diagnosis, and bilateral^3^; factors all associated with implant-based reconstruction. Studies have previously identified that implant-based reconstruction has a higher rates of re-operation and complications, incidence up to 46%.^4-7^

A well-established method for breast reconstruction is 2-stage reconstruction, whereby an expander is initially inserted immediately or in a delayed fashion, followed by the placement of an implant after a period of expansion.^8^ Two-stage reconstruction with a tissue expander/implant (TE/I) technique remains the most common breast reconstructive approach following mastectomy.^9-11^ The American Society of Plastic Surgery 2020 Annual Report showed that of the 137,808 breast reconstructions, 105,665 (77%) were immediate and 83,487 (61%) used TE/I as a prothesis.^12^ The use of acellular dermal matrix (ADM) may be warranted for soft tissue support. Its use^13^ and type^14,15^ been investigated as independent risk factors for complications.

One-stage and 2-stage reconstruction was previously compared with no significant difference in complications or re-operation rates.^16^ Our center's experience investigating a cohort of TE/I patients provides an opportunity to critically evaluate outcomes independent of ADM use and identify patient-related risk factors for complications in a Canadian context. As such, the primary objective of this study was to analyze the incidence of complications experienced by 2-stage breast reconstruction patients. The secondary objectives of this study were to (1) report the types of major and minor complications; (2) explore risk factors for these complications; and (3) document the experience and burden of tissue expansion.

## Methods

This study is reported in accordance with The Strengthening the Reporting of Observational Studies in Epidemiology (STROBE) Statement.^17^

### Study Design and Setting

This was a retrospective chart review study. Patients that underwent breast reconstruction using the 2-stage TE/I technique at a single institution were identified using Ontario Health Insurance Plan billing codes. Ethics approval was obtained (REB #12773).

### Patients

Charts of patients that underwent post-mastectomy, 2-stage TE/I-based breast reconstruction without the use of ADM were reviewed. These cases were performed between 2016 and 2019. Inclusion criteria were: (1) women 18 years of age or older at the time of reconstruction; and (2) primary reconstruction with TEs, with or without latissimus dorsi. Exclusion criteria were: (1) direct-to-implant reconstruction; (2) combined autologous and alloplastic reconstruction; (3) not a primary reconstruction; and (4) ADM used.

### Variables and Data Sources

Patient data were collected from electronic medical records and inputted into the Research Electronic Data Capture (REDCap) application by a research team member. Data for demographics and cancer treatment (age, BMI, smoking status, radiation, chemotherapy), complications (number and type, treatment of complication, classification of complication), tissue expansion information (date of insertion, date of exchange or removal, number of fills, volume of fills and total volume of fills prior to exchange), and implant characteristics (type, brand, size, etc) were recorded. The occurrence of any major (re-operation, readmission, loss of device) or minor surgical complications (infection, asymmetry, dehiscence etc) were recorded for the time horizon from the date of mastectomy until 3 months following the date of TE exchange surgery to capture acute complications.

### Statistical Analysis

The data were presented as mean (standard deviation), median (range), or number (percentage). Patients were used as the unit of analysis. Student's *t*-test and Chi-Square analysis were used to compare continuous and categorical variables, respectively to determine whether there was a statistically significant difference between patients with and without complications. A univariate analysis was used to determine which variables were significant predictors for complications. A multivariate logistic regression with stepwise selection and the rule of 10 events per variable were used to develop the regression model. To account for heterogeneity confounding variables were entered into the model and breasts were used as the unit of analysis. All statistical tests used 2-sided hypotheses with *P*-values less than .05 considered statistically significant. Statistical analyses were performed using R Software (R Studio, Vienna Austria).

## Results

### Participant Characteristics

During the study period, 55 patient charts were identified. Patient characteristics are presented in Table 1. Mean age at reconstruction was 49.0 (SD 9.9) years. Mean BMI at reconstruction was 27.3 (SD 6.4) kg/m2. Twenty-one patients (38%) were previous or current smokers and 19 patients (35%) received radiation therapy. About 51% of patients (n = 28) underwent mastectomy for unilateral cancer with prophylactic surgery on the contralateral side. There were 19 (35%) unilateral and 36 (66%) bilateral reconstructions. A total of 91 TE/Is were placed, with most TEs (n = 59, 65%) placed immediately. All implants were placed in a submuscular pocket without ADM. Implant characteristics are presented in Table 2. Mean volume of implants ultimately placed was 493 (SD = 156.7) cc. Most implants were silicone (n = 88, 97%), smooth (n = 86, 95%), and round (n = 85, 93%).

**Table 1.** Demographic and Clinical Characteristics of Breast Reconstruction Patients.

Variable	Total (n = 55)	Patients without complications (n = 30)	Patients with complications (n = 25)
**Mean age at reconstruction, years**	49.0 ± 9.9	49.3 ± 11.2	50.1 ± 9.3
**Mean BMI at reconstruction, kg/m^2^**	27.3 ± 6.4	25.5 ± 3.4	28.4 ± 6.5
**Smoking status, n (%)**			
Never	33 (61.1)	19 (63.3)	14 (56.0)
Current	8 (14.8)	6 (20.0)	7 (28.0)
Previous	13 (24.1)	3 (10.0)	5 (20.0)
**Chemotherapy, n (%)**			
Prior to reconstruction	14 (25.9)	7 (23.3)	7 (28.0)
Post reconstruction	6 (11.1)	2 (6.7)	4 (16.0)
No chemotherapy	34 (63.0)	21 (70.0)	15 (60.0)
**Radiation, n (%)**			
Prior to reconstruction	14 (25.9)	4 (13.3)	10 (40.0)
Post reconstruction	5 (9.3)	1 (3.3)	4 (16.0)
No radiation	35 (64.8)	25 (83.3)	12 (48.0)
**Mastectomy purpose, n (%)**			
Unilateral cancer	20 (36.4)	12 (40.0)	8 (32.0)
Bilateral cancer	1 (1.8)	0 (0.0)	1 (4.0)
Unilateral cancer and contralateral prophylactic	28 (50.9)	13 (43.3)	15 (60.0)
Double prophylactic	6 (10.9)	4 (13.3)	2 (8.0)
**Reconstruction side, n (%)**			
Unilateral right	11 (12.1)	8 (26.7)	3 (12.0)
Unilateral left	8 (14.8)	3 (10.0)	5 (20.0)
Bilateral	36 (65.5)	18 (60.0)	18 (72.0)
**Breasts as unit**	**Total breasts** **(n = 91)**	**Breasts without complications** **(n = 47)**	**Breasts with complications** **(n = 44)**
**Reconstruction type**			
TE to implant	80 (87.9)	41 (87.2)	39 (88.6)
LD and TE to implant	11 (12.1)	6 (12.8)	5 (11.4)
**Reconstruction timing**			
Immediate	59 (64.8)	25 (53.2)	34 (77.3)
Delayed	32 (35.2)	22 (46.8)	10 (22.7)

Unit of analysis is the patient.

**Table 2.** Implant Characteristics.

Variable	Implant n = 91(%)
**Mean volume (cc)**	493 ± 156.7
**Brand, n (%)**	
Allergan	33 (36.3)
Mentor	55 (60.4)
Not reported	3 (3.3)
**Type, n (%)**	
Silicone	88 (96.7)
Saline	3 (3.3)
**Projection, n (%)**	
Low	2 (2.2)
Moderate	6 (6.6)
moderate plus	3 (3.3)
High	62 (6.8)
Ultra-high	12 (13.2)
Not reported	6 (6.6)
**Surface, n (%)** Smooth	85 (93.4)
Textured	2 (2.2)
Not reported	4 (4.4)
**Shape, n (%)**	
Round	85 (93.4)
Anatomic	2 (2.2)
Not reported	4 (4.4)

### Primary Outcome

Twenty-five patients (45%) had at least 1 complication. Complication characteristics for patients are presented in Table 3. The mean complication per patient was 0.84 (SD = 1.2), with 18 patients requiring admission (72%) and 17 patients requiring revision surgery (68%).

**Table 3.** Complication Characteristics Presented by Frequency, Location, and Classification.

Variable	n = 25 (%)
**Mean complications per patient**	0.84 ± 1.2
**Complications requiring readmission**	18 (72.0)
**Complications requiring revision surgery**	17 (68.0)

Patients as unit of analysis.

### Secondary Outcomes

**Figure 1.**
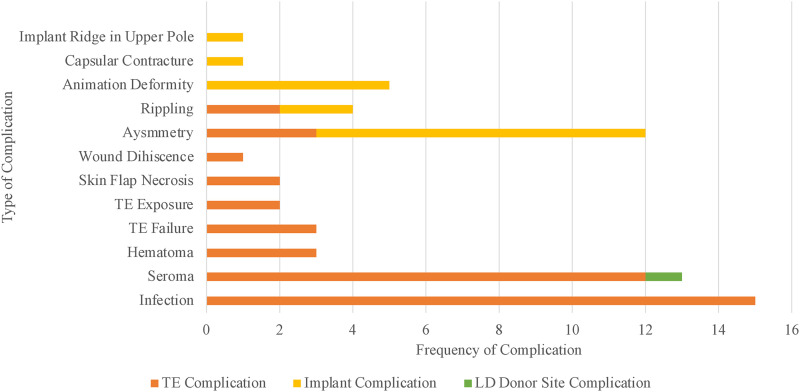
Complications presented by frequency and source with breast as the unit of measurement.

Abbreviation: TE, tissue expander.

The frequency and source of complications for all patients are presented in Figure 1. Of the patients with complications, 17 (68%) had only 1 complication post-operatively. Immediate reconstruction had the highest incidence of complications (n = 20, 80%) for timing of reconstruction. The most common reported complication in patients was infection associated with the TE (n = 10, 40%). There was 1 seroma (4%) that was associated with latissimus dorsi donor site. Asymmetry was the most common complication associated with implants in patients (n = 5, 20%).

**Figure 2.**
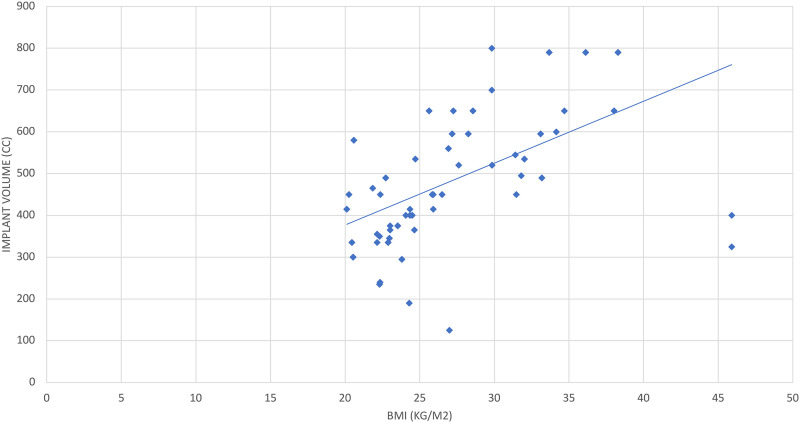
Relationship between BMI and implant volume (breasts as the unit of analysis).

The mean number of fill appointments was 3.6 ± 1.7 (range: 1-8). The correlation coefficient between BMI and implant volume (cc) was *r* = 0.61, indicating a moderate positive correlation (Figure 2). Univariate linear regression demonstrated that for every increase in BMI, there was a 14.8 cc increase in implant volume, on average (*P* < .001).

Univariate analysis to identify potential predictors of complications associated with 2-stage TE/I reconstruction is presented in Table 4. Significant predictors of a complication were BMI (β= 1.11 [95% CI: 1.00-1.24], *P* = .047) and radiation therapy (β= 0.19 [95% CI: 0.54-0.64], *P* = .008). These significant variables, as well as clinically significant variables were used to build a multivariate logistic regression model (Table 5). Radiation and BMI were still found to be significant predictors in the multivariate model (β= 1.17 [95% CI: 1.05-1.56], *P* = .049 and β= 0.08 [95% CI: 0.01-0.84], *P* = .036, respectively).

**Table 4.** Univariate Analysis and Potential Confounders of Complications.

Variable	Exp (B)	95% CI	*P*-value
**Age, years** (continuous)	1.01	(0.95-1.06)	.820
**BMI, kg/m^2^** (continuous)	1.11	(1.00-1.24)	.**047**
**Smoking status** (past and present vs non-smoker)	1.81	(0.60-5.47)	.293
**Radiation** (yes vs no)	5.37	(1.56-18.49)	.**008**
**Number of fills** (continuous)	0.97	(0.71-1.34)	.867
**Implant volume (cc)** (continuous)	1.00	(0.99-1.00)	.698

Breasts as unit of analysis.

Bold indicates *p*-values are significant.

**Table 5.** Multivariate Logistics Regression Analysis of Predictive Variables for Complications.

Variable	Exp (B)	95% CI	*P*-value
**Age, years** (continuous)	0.98	(0.88-1.09)	.728
**BMI, kg/m^2^** (continuous)	1.17	(1.05-1.56)	.**049**
**Smoking status** (past and present vs non-smoker)	1.44	(0.23-8.91)	.695
**Radiation** (yes vs no)	6.67	(1.19-12.64)	.**036**
**Number of fills** (continuous)	0.94	(0.53-1.65)	.867
**Implant volume (cc)** (continuous)	0.99	(0.99-1.00)	.774

Breasts as the unit of analysis.

Bold indicates *p*-values are significant.

## Discussion

This retrospective cohort study provides a complication profile for a Canadian, single-center experience with 2-stage TE/I. The results demonstrated an association between BMI and complications, which is supported by the literature;^18^ combined with the number of appointments, this information can be used to counsel overweight and obese patients.^5,7^ Additionally, this study supports the literature regarding the higher incidence of revision surgery following 2-stage TE/I reconstruction.^19,20^ The timing of this cohort includes patients undergoing reconstruction with total submuscular technique; all complications were independent of ADMs. The utilization ADM as an adjunct in breast reconstruction, particularly in direct-to-implant, has exhibited notable trends in recent years.^21^ Despite its benefits, the use of ADM in breast reconstruction is associated with a higher incidence of infection, seroma formation, and reconstruction failure.^13,22,23^

Several cohort studies have examined complication and revision surgery for 2-stage reconstruction.^6,24-29^ Data has only been compared for direct-to-implant and TE/I reconstruction in large registry studies, as there are no published randomized controlled trials for head-to-head comparisons. A Dutch Breast Implant Registry of 3948 patients reported that direct-to-implant compared to 2-stage TE/I reconstruction had a lower incidence of revision surgery (4.0% vs 11.7%), but higher complication rates (19% vs 16%).^30^ In contrast, an Australian Breast Device Registry with 5152 breast reconstructions found that direct-to-implant reconstruction had a higher incidence of complications compared to TE/I reconstruction (61.5% vs 55.3%; *P* < .001) as well as a higher all cause revision incidence (10.9% vs 5.5% at 12 months and 24.4% vs 14.4% at 48 months).^31^ Although both direct-to-implant and TE/I have been associated with complications and revision surgeries, the decision-making process between these 2 types of reconstruction is multifactorial and requires a personalized conversation with patients as direct comparisons due to lack of high-quality studies.

This study is not without limitations. First, due to the retrospective nature of the study, selection bias may be present.^32^ Second, as this was a single-center experience, it inherently has a small sample size. Sample size was not calculated *a priori*, however, the regression analysis rule of events per variable was used to ensure the analysis was adequately powered.^33^ Although inclusion criteria were kept broad, it is possible that the breast cancer population at our center may differ from others. Third, the patient's pathology was not taken into consideration for the analysis and may have affected their prognostic and complication profiles. Finally, patients were only followed up to 3 months post-reconstruction. As such, patients may have developed complications past this time horizon, or had repeated intervention for their index complication.

In conclusion, this is the first Canadian study to investigate complication profiles of 2-stage TE/I for breast reconstruction following mastectomy. Infection remains to be the leading cause of complications. BMI and radiation are significant predictors of complications. Larger prospective multicenter trials may elicit nuanced variation among different population demographics.
